# Supplementary material for: Impact of combined pulmonary fibrosis and emphysema on lung cancer risk and mortality in rheumatoid arthritis: A multicenter retrospective cohort study
Source: PLoS One. 2024 Feb 27;19(2):e0298573. doi: 10.1371/journal.pone.0298573 (PMC10898759; doi:10.1371/journal.pone.0298573)
Supplement: S1 Checklist — (DOCX) [file pone.0298573.s001.docx]

STROBE Statement—checklist of items that should be included in reports of observational studies

|  | Item No. | Recommendation | Page  No. | Relevant text from manuscript |
| --- | --- | --- | --- | --- |
| **Title and abstract** | 1 | (*a*) Indicate the study’s design with a commonly used term in the title or the abstract | Pages 1 and 3 | Lines 2–4 and 43–45 |
|  |  | (*b*) Provide in the abstract an informative and balanced summary of what was done and what was found | Pages 3 and 4 | Lines 37–61 |
| Introduction | | | |  |
| Background/rationale | 2 | Explain the scientific background and rationale for the investigation being reported | Pages 5 and 6 | Lines 63–96 |
| Objectives | 3 | State specific objectives, including any prespecified hypotheses | Page 6 | Lines 97–106 |
| Methods | | | |  |
| Study design | 4 | Present key elements of study design early in the paper | Pages 7 and 8 | Lines 125–147 |
| Setting | 5 | Describe the setting, locations, and relevant dates, including periods of recruitment, exposure, follow-up, and data collection | Pages 6–8 | Lines 109–123 and 144–147 |
| Participants | 6 | (*a*) *Cohort study*—Give the eligibility criteria, and the sources and methods of selection of participants. Describe methods of follow-up  *Case-control study*—Give the eligibility criteria, and the sources and methods of case ascertainment and control selection. Give the rationale for the choice of cases and controls  *Cross-sectional study*—Give the eligibility criteria, and the sources and methods of selection of participants | Pages 6–8 | Lines 109–123 and 144–147 |
|  |  | (*b*) *Cohort study*—For matched studies, give matching criteria and number of exposed and unexposed  *Case-control study*—For matched studies, give matching criteria and the number of controls per case | Not applicable | – |
| Variables | 7 | Clearly define all outcomes, exposures, predictors, potential confounders, and effect modifiers. Give diagnostic criteria, if applicable | Page 8 | Lines 135–147 |
| Data sources/ measurement | 8* | For each variable of interest, give sources of data and details of methods of assessment (measurement). Describe comparability of assessment methods if there is more than one group | Pages 7–9 | Lines 126–134, 149–155, 157-171, and 173–177 |
| Bias | 9 | Describe any efforts to address potential sources of bias | Pages 7–9 | Lines 126–134 and 158–162 |
| Study size | 10 | Explain how the study size was arrived at | Page 9 | Lines 179–181 |

Continued on next page

| Quantitative variables | 11 | Explain how quantitative variables were handled in the analyses. If applicable, describe which groupings were chosen and why | Not applicable | – |
| --- | --- | --- | --- | --- |
| Statistical methods | 12 | (*a*) Describe all statistical methods, including those used to control for confounding | Pages 10 and 11 | Lines 195–226 |
|  |  | (*b*) Describe any methods used to examine subgroups and interactions | Not applicable | – |
|  |  | (*c*) Explain how missing data were addressed | Page 10 | Lines 201 and 202 |
|  |  | (*d*) *Cohort study*—If applicable, explain how loss to follow-up was addressed  *Case-control study*—If applicable, explain how matching of cases and controls was addressed  *Cross-sectional study*—If applicable, describe analytical methods taking account of sampling strategy | Page 11 | Lines 206–222 |
|  |  | (*e*) Describe any sensitivity analyses | Not applicable | – |
| Results | | | | |
| Participants | 13* | (a) Report numbers of individuals at each stage of study—eg numbers potentially eligible, examined for eligibility, confirmed eligible, included in the study, completing follow-up, and analysed | Pages 12–14 | Lines 230–232, 257–265, and 274–280 |
|  |  | (b) Give reasons for non-participation at each stage | Pages 13 and 14 | Lines 257–265 and 274–280 |
|  |  | (c) Consider use of a flow diagram | Not applicable | – |
| Descriptive data | 14* | (a) Give characteristics of study participants (eg demographic, clinical, social) and information on exposures and potential confounders | Pages 11–13  Table 1  S1 Table | Lines 229–254  Lines 618–627  Supporting Information File |
|  |  | (b) Indicate number of participants with missing data for each variable of interest | Page 12 | Line 232 |
|  |  | (c) *Cohort study*—Summarise follow-up time (eg, average and total amount) | Pages 13  Table 2  Table 3 | Lines 257 and 258  Lines 628–635  Lines 636–647 |
| Outcome data | 15* | *Cohort study*—Report numbers of outcome events or summary measures over time | Pages 13 and 14  Table 2  Table 3 | Lines 258–265 and 274–280  Lines 628–635  Lines 636–647 |
|  |  | *Case-control study—*Report numbers in each exposure category, or summary measures of exposure |  |  |
|  |  | *Cross-sectional study—*Report numbers of outcome events or summary measures |  |  |
| Main results | 16 | (*a*) Give unadjusted estimates and, if applicable, confounder-adjusted estimates and their precision (eg, 95% confidence interval). Make clear which confounders were adjusted for and why they were included | Pages 13–15  Table 4  S2 Table  Figure 1  Figure 2 | Lines 266–271, 281–291, and 293–310  Lines 648–658  Supporting Information File  Lines 598–603  Lines 605–617 |
|  |  | (*b*) Report category boundaries when continuous variables were categorized | Not applicable | – |
|  |  | (*c*) If relevant, consider translating estimates of relative risk into absolute risk for a meaningful time period | Pages 13 and 14  Table 2  Table 3 | Lines 263–265 and 276–280  Lines 628–635  Lines 636–647 |

| Other analyses | 17 | Report other analyses done—eg analyses of subgroups and interactions, and sensitivity analyses | Not applicable | – |
| --- | --- | --- | --- | --- |
| Discussion | | | | |
| Key results | 18 | Summarise key results with reference to study objectives | Page 15 | Lines 313–322 |
| Limitations | 19 | Discuss limitations of the study, taking into account sources of potential bias or imprecision. Discuss both direction and magnitude of any potential bias | Page 18 | Lines 382–396 |
| Interpretation | 20 | Give a cautious overall interpretation of results considering objectives, limitations, multiplicity of analyses, results from similar studies, and other relevant evidence | Pages 15–19 | Lines 323–381 and 398–407 |
| Generalisability | 21 | Discuss the generalisability (external validity) of the study results | Page 18 | Lines 394–396 |
| Other information | |  | | |
| Funding | 22 | Give the source of funding and the role of the funders for the present study and, if applicable, for the original study on which the present article is based | Page 20 | Lines 408–415 |

*Give information separately for cases and controls in case-control studies and, if applicable, for exposed and unexposed groups in cohort and cross-sectional studies.

**Note:** An Explanation and Elaboration article discusses each checklist item and gives methodological background and published examples of transparent reporting. The STROBE checklist is best used in conjunction with this article (freely available on the Web sites of PLoS Medicine at http://www.plosmedicine.org/, Annals of Internal Medicine at http://www.annals.org/, and Epidemiology at http://www.epidem.com/). Information on the STROBE Initiative is available at www.strobe-statement.org.
