# Supplementary figures and images for: Impact of combined pulmonary fibrosis and emphysema on lung cancer risk and mortality in rheumatoid arthritis: A multicenter retrospective cohort study
Source: PLoS One. 2024 Feb 27;19(2):e0298573. doi: 10.1371/journal.pone.0298573 (PMC10898759; doi:10.1371/journal.pone.0298573)

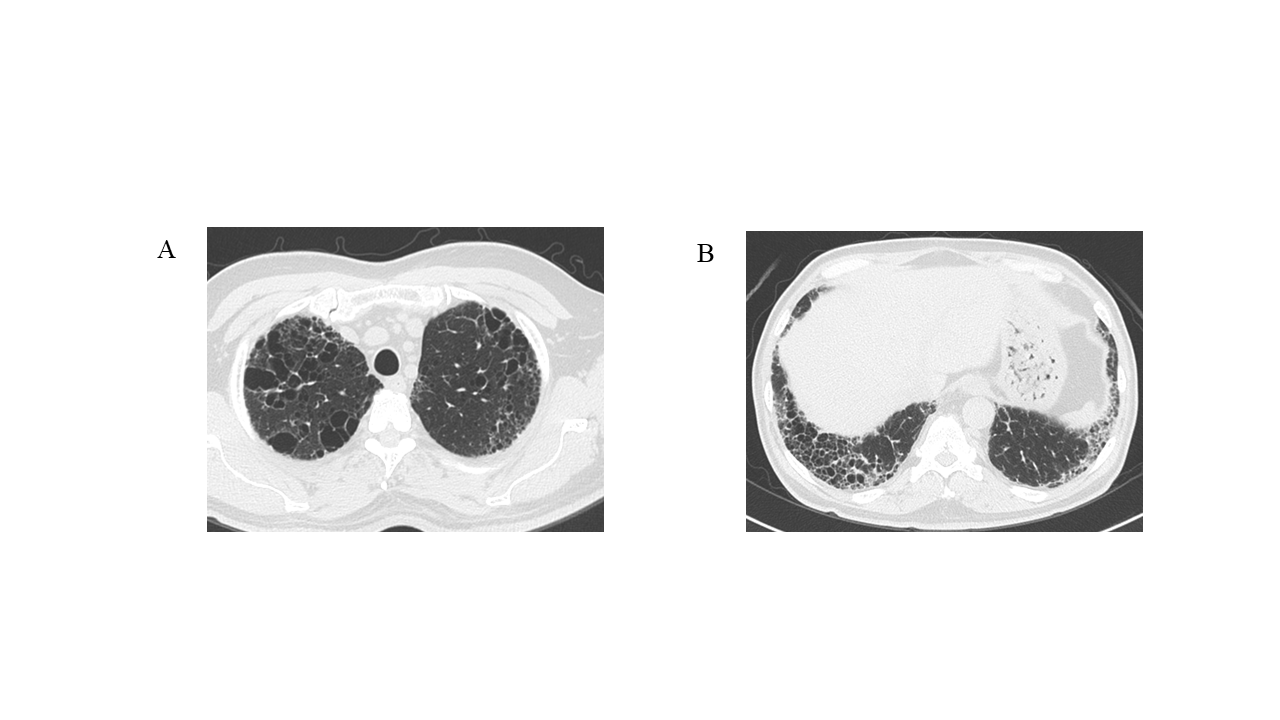

Supplement: S1 Fig — HRCT images show the coexistence of emphysema (A) and pulmonary fibrosis (B, the UIP pattern of fibrotic ILD). The images show a typical distribution of the disease in CPFE. The zones of fibrosis and emphysema are completely separated. Paraseptal emphysema is localized to the upper lobes and fibrosis characterized by honeycombing and traction bronchiectasis is localized to the lung bases. Reticular abnormality is also present. HRCT, high-resolution computed tomography; RA, rheumatoid arthritis; CPFE, combined pulmonary fibrosis and emphysema; UIP, usual interstitial pneumonia; ILD, interstitial lung disease. (TIF) [file pone.0298573.s002.tif]
