# Supplementary material for: Impact of combined pulmonary fibrosis and emphysema on lung cancer risk and mortality in rheumatoid arthritis: A multicenter retrospective cohort study
Source: PLoS One. 2024 Feb 27;19(2):e0298573. doi: 10.1371/journal.pone.0298573 (PMC10898759; doi:10.1371/journal.pone.0298573)
Supplement: S1 Table — (DOCX) [file pone.0298573.s003.docx]

**S1 Table. DMARD therapy at diagnosis of lung cancer**

|  | RA patients |
| --- | --- |
| DMARD therapy for RA,^†^ number (%) | (n = 82) |
| MTX monotherapy | 32 (39.0) |
| MTX plus bDMARD or JAK inhibitor | 6 (7.3) |
| MTX plus other csDMARD^‡^ | 3 (3.7) |
| TNF inhibitor monotherapy | 4 (4.9) |
| IL-6 inhibitor monotherapy | 5 (6.1) |
| JAK inhibitor monotherapy | 4 (4.9) |
| Other csDMARD monotherapy^‡^ | 22 (26.8) |
| No DMARD use^§^ | 6 (7.3) |

^†^DMARD therapy was discontinued following the diagnosis of lung cancer.

**^‡^**Other csDMARDs included salazosulfapyridine, bucillamine, mizoribine, cyclosporin, and tacrolimus.

^§^Among these 6 patients, 5 were receiving PSL at the diagnosis of lung cancer and 1 was not treated for RA.

RA, rheumatoid arthritis; DMARD, disease-modifying antirheumatic drug; bDMARD, biological DMARD; csDMARD, conventional synthetic DMARD; MTX, methotrexate; TNF, tumor necrosis factor; IL-6, interleukin-6; JAK, Janus kinase; PSL, prednisolone.
