# Supplementary material for: Impact of combined pulmonary fibrosis and emphysema on lung cancer risk and mortality in rheumatoid arthritis: A multicenter retrospective cohort study
Source: PLoS One. 2024 Feb 27;19(2):e0298573. doi: 10.1371/journal.pone.0298573 (PMC10898759; doi:10.1371/journal.pone.0298573)
Supplement: S2 Table — (DOCX) [file pone.0298573.s004.docx]

**S2 Table. Comparison of RA-related factors among lung cancer patients grouped according to HRCT-based CPFE diagnosis**

|  | CPFE | ILD or emphysema | Without ILD or emphysema | *p*-value* |
| --- | --- | --- | --- | --- |
|  | (n = 33) | (n = 27) | (n = 22) |  |
| RA duration, years, mean (SD) | 10.9 (9.5) | 12.3 (10.4) | 11.0 (12.9) | 0.94 |
| RA activity, low or remission, number (%) | 20 (60.6) | 20 (74.1) | 16 (72.7) | 0.47 |
| Steinbrocker stage III/IV, number (%) | 11 (33.3) | 10 (37.0) | 9 (40.9) | 0.85 |
| Desaturation with exercise (SpO_2_ <90%), number (%) | 28 (84.8) | 11 (40.7) | 0 | <0.001 |

*Differences between patient groups were assessed using ANOVA for continuous variables and Fisher’s exact probability test for categorical variables.

RA, rheumatoid arthritis; HRCT, high-resolution computed tomography; SpO_2_, oxygen saturation as measured using pulse oximeter; CPFE, combined pulmonary fibrosis and emphysema; ILD, interstitial lung disease; ANOVA, analysis of variance; SD, standard deviation.
