# Supplementary material for: Impact of combined pulmonary fibrosis and emphysema on lung cancer risk and mortality in rheumatoid arthritis: A multicenter retrospective cohort study
Source: PLoS One. 2024 Feb 27;19(2):e0298573. doi: 10.1371/journal.pone.0298573 (PMC10898759; doi:10.1371/journal.pone.0298573)
Supplement: S3 Table — (DOCX) [file pone.0298573.s005.docx]

**S3 Table.** **Predictive factors for lung cancer-related death in RA patients newly diagnosed with lung cancer (Model 1)**

|  | Unadjusted HR | *p-*value | Adjusted HR | *p-*value |
| --- | --- | --- | --- | --- |
|  | (95% CI) |  | (95% CI) |  |
| Age per additional year | 1.05 (1.01⎼1.09) | 0.008 | 1.02 (0.98⎼1.05) | 0.28 |
| Male vs. female | 1.58 (0.90⎼2.78) | 0.11 | – | – |
| RA duration per additional year | 1.02 (1.00⎼1.03) | 0.12 | – | – |
| RA activity: |  |  |  |  |
| Low/remission vs. high/moderate | 0.64 (0.37–1.13) | 0.12 |  |  |
| Steinbrocker stages III/IV vs. I/II | 1.23 (0.72⎼2.10) | 0.46 | – | – |
| Smoking history ≥30 PYs | 1.50 (0.86⎼2.62) | 0.15 | – | – |
| HRCT-based CPFE diagnosis |  |  |  |  |
| CPFE | 2.76 (1.36⎼5.59) | 0.005 | 2.14 (1.06⎼4.31) | 0.030 |
| ILD alone or emphysema alone | 1.27 (0.59⎼2.73) | 0.54 | 1.33 (0.57⎼3.11) | 0.51 |
| Without ILD or emphysema | 1 (reference) | ⎼ | 1 (reference) | – |
| Histological type of lung cancer |  |  |  |  |
| Adenocarcinoma | 1 (reference) | ⎼ | – | – |
| Squamous cell carcinoma | 1.23 (0.66⎼2.29) | 0.51 | – | – |
| Small cell carcinoma | 1.90 (0.84⎼4.31) | 0.13 | – | – |
| TNM clinical stage of lung cancer |  |  |  |  |
| Stage I | 1 (reference) | ⎼ | 1 (reference) | – |
| Stage II | 4.08 (1.97⎼8.47) | <0.001 | 2.58 (1.02⎼6.50) | 0.045 |
| Stage III | 3.46 (1.45⎼8.25) | 0.001 | 1.92 (0.78⎼4.73) | 0.16 |
| Stage IV | 6.96 (3.28⎼14.76) | <0.001 | 2.31 (0.78⎼6.86) | 0.13 |
| Surgery for lung cancer^†^ |  |  |  |  |
| Yes vs. no | 0.15 (0.08⎼0.29) | <0.001 | 0.32 (0.14⎼0.71) | 0.005 |

Univariable and multivariable Fine-Gray competing risks regression analyses were conducted to evaluate baseline patient characteristics that predict lung cancer-related death outcome after lung cancer diagnosis. All predictor variables with *p-*values <0.10 in the univariable models were introduced into multivariable analysis with forced entry procedure. In this model, desaturation with exercise (SpO_2_ <90% during a 6-minute walk) was not included in the multivariable regression analysis with HRCT-based CPFE diagnosis because both predictor variables were highly correlated.

^†^Defined as surgery that was performed as the initial treatment within 1 month after the diagnosis of lung cancer.

RA, rheumatoid arthritis; PY, pack-year; HRCT, high-resolution computed tomography; CPFE, combined pulmonary fibrosis and emphysema, ILD, interstitial lung disease; TNM, tumor, node, and metastasis; HR, hazard ratio; 95% CI, 95% confidence interval; SpO_2_, oxygen saturation as measured using pulse oximeter.
