# Supplementary material for: Impact of combined pulmonary fibrosis and emphysema on lung cancer risk and mortality in rheumatoid arthritis: A multicenter retrospective cohort study
Source: PLoS One. 2024 Feb 27;19(2):e0298573. doi: 10.1371/journal.pone.0298573 (PMC10898759; doi:10.1371/journal.pone.0298573)
Supplement: Attachment — Submitted filename: Response to Reviewers.docx [file pone.0298573.s007.docx]

**Response to Reviewers**

We are most grateful to the reviewers for their valuable comments as well as the time and energy spent for our manuscript. We have made the requested changes and added new information to the manuscript in response to their insightful comments. All alterations are highlighted in red text in the revised manuscript. We are confident that the manuscript has benefited from the reviewers’ useful comments and suggestions.

Below are point-by-point replies to the reviewers’ comments.

**Reply to Reviewer 1**

We wish to express our appreciation to the reviewer for the comment regarding our study. We understand that it is important to provide novel insights into the role of combined pulmonary fibrosis and emphysema (CPFE) in lung cancer pathogenesis. However, we must emphasize that this is the first cohort study to examine the impact of CPFE on lung cancer risk and lung cancer-related mortality in patients with rheumatoid arthritis (RA). RA is a chronic inflammatory disease, and the high prevalence of interstitial lung disease (ILD) in RA is well known. Additionally, the higher risk of lung cancer in RA patients compared with that in the general population has been reported previously. Recently, several risk factors for lung cancer in RA, such as male sex, smoking, and RA-associated ILD, have been proposed. RA-associated CPFE should be considered an entity distinct from RA-associated ILD. Nevertheless, few clinical studies have examined possible associations between RA-associated CPFE and lung cancer risk and mortality. First, we need to obtain clinical evidence for these associations among RA patients, then we can move forward to address the underlying molecular mechanisms in the CPFE-associated pathogenesis of lung cancer in RA. In 2022, the research definition and classification criteria of CPFE as a clinical syndrome were formalized in an official statement from the American Thoracic Society (ATS), the European Respiratory Society (ERS), Japanese Respiratory Society (JRS), and the Latin American Thorax Association (ALAT). Using this new definition of CPFE, we conducted the present multicenter cohort study. To clarify these points, we have revised the title, Abstract, Introduction, and Discussion sections (lines 2, 3, 30, 33–36, 76–99, and 364–366). New references were added in the revised version (Refs. 23, 26, and 28).

1. We thank the reviewer for the comment regarding high-resolution computed tomography (HRCT) images of CPFE and ILD. In response, we have included figures showing pulmonary HRCT scans of emphysema and ILD (UIP pattern) in an RA patient who was diagnosed with CPFE. The images show the typical distribution of the disease in CPFE. Paraseptal emphysema is localized to the upper lobes. Fibrosis, characterized by honeycombing and traction bronchiectasis, is localized to the lower lobes in a background of reticulation. These findings are consistent with the official ATS/ERS/JRS/ALAT clinical practice guideline. We included these HRCT images as S1 Fig in the revised manuscript (S1 Fig; lines 256–257 and 499–508).
2. We appreciate the reviewer’s comment regarding biopsy data from CPFE and ILD. This cohort study was performed retrospectively in real-world clinical settings for RA and non-RA patients who were diagnosed with lung cancer between June 2006 and December 2021. Pulmonary HRCT images and histological data of lung cancer were obtained from all participants. Unfortunately, lung biopsy samples from fibrosis lesions were available for only a few patients with CPFE or ILD. We understand that lung biopsy is the gold standard for the classification of UIP patterns. For patients with HRCT findings suggestive of a diagnosis other than IPF, histological examinations might have been useful to exclude other known causes of pulmonary fibrosis. In many cases, however, the treating physicians did not perform lung biopsy because of concerns about the risk of biopsy-related complications. The risk of lung biopsy could outweigh the benefit of establishing a firm diagnosis of the UIP. For patients with RA-associated ILD, histological confirmation was not required because RA was definitively diagnosed as the cause of ILD. In addition, the utility of detailed histopathological scoring systems in day-to-day clinical management of patients with pulmonary fibrosis has not been evaluated. For this study, we collected HRCT images taken at the time of lung cancer diagnosis from all participants, which were viewed in random order and independently by three experts (a board-certified radiologist and two board-certified pulmonologists). We are confident with the HRCT-based diagnoses in this study.

We agree with the reviewer’s suggestion that it would be valuable to examine the number of fibroblast foci and immunohistochemistry biomarkers, as well as their associations with lung cancer-related mortality. Regrettably, however, for the above-mentioned reasons, we were unable to do such histological analyses for pulmonary fibrosis in ILD or CPFE patients. Needless to say, at the time of lung cancer diagnosis, treating physicians performed lung biopsies for all patients and obtained small tumor samples, because pathological classification of lung cancer is essential for optimal therapeutic strategies. However, lung biopsy was not performed for non-tumor lesions at that time for ethical reasons based on the above-mentioned risks and benefits.

We included this lack of histopathological data in the Discussion section as one of the limitations of this study in the revised manuscript (lines 425–430).

3. We are grateful for the reviewer’s suggestion regarding plasma markers. We understand that there are various plasma biomarkers for lung cancer. However, their sensitivity and specificity for lung cancer diagnosis are not sufficiently high for routine clinical use. Moreover, a uniform serum biomarker composition capable of distinguishing lung cancer types has yet to be discovered. Therefore, the treating physicians at our institutions did not measure levels of plasma biomarkers at the time of lung cancer diagnosis. As the reviewer suggested, it would be interesting to evaluate the prognostic utility of plasma biomarkers for lung cancer mortality. However, it was beyond the scope of the present study. We described the need for further studies to address which biomarkers could be used to predict outcomes in RA patients with lung cancer and CPFE in the Discussion section (lines 430–432).
4. We thank the reviewer for the suggestion to examine the association between CPFE and drug response. Regarding cancer treatment, as we described in the Discussion section as a limitation, we did not include the type and implementation of cancer treatment used during follow-up into survival analysis as predictor variables because they were time-varying covariates. Instead, we adopted surgery performed within 1 month after cancer diagnosis for survival analysis (lines 412–415). Non-surgical cancer treatment included chemotherapy and radiation therapy, which are well known to exacerbate pulmonary fibrosis in ILD and CPFE. Treating physicians did not administer chemotherapy in the majority of ILD or CPFE patients due to concerns about this complication. Accordingly, we were unable to examine the effect of ILD or CPFE on response to anticancer drugs. We added this information to the Results section (lines 269–270).

Regarding disease-modifying antirheumatic drugs (DMARDs), as we listed in S1 Table, a number of DMARDs were used before the diagnosis of lung cancer (methotrexate, biological DMARDs, Janus kinase inhibitors, salazosulfapyridine, bucillamine, mizoribine, cyclosporin, and tacrolimus) as well as prednisolone. Given the role of the immune system in tumor surveillance, immunosuppressive or immunomodulatory therapies for RA could be implicated in the risk of malignancy. Therefore, DMARD use at the time of lung cancer diagnosis was not included in the survival analysis because these drugs were discontinued during cancer treatment due to concerns about tumor recurrence. We address this point in the Discussion section as a limitation (lines 422–425). A new reference was added in the revised version (Ref. 38).

Please understand that this was a retrospective observational study in real-world clinical settings. We could not use therapeutic strategies that were not considered beneficial to patients.

5. We agree with the reviewer’s suggestion to discuss how our findings can improve the current management of lung cancer in RA patients with CPFE. In the present study, CPFE was more frequently observed in RA patients than in non-RA patients at the time of lung cancer diagnosis. Considering a possible contribution of idiopathic CPFE to the risk of lung cancer development, the higher prevalence of CPFE in RA patients may, at least in part, explain the increased risk of lung cancer in RA patients compared with the general population. Mass screening of all RA patients using HRCT is not supported by current evidence, but thorough clinical evaluation and regular screening for lung cancer with imaging are needed in RA patients with CPFE, which should enable early diagnosis of lung cancer and better prognosis. We also found that RA patients with CPFE had significantly increased cancer-related mortality compared with those without ILD or emphysema. The risk of acute exacerbation of ILD is of particular concern in CPFE patients after surgical resection, radiation, and chemotherapy. Limited options for lung cancer treatment and treatment-associated complications may explain the increased lung cancer-related mortality in this patient population. It is necessary to evaluate surgical tolerance and to closely monitor pulmonary conditions during lung cancer treatment in RA patients with CPFE. To clarify these points, we added one paragraph in the Discussion section of the revised version (lines 397–411). In addition, several sentences were added to the Abstract and Discussion (lines 58–60, 384–386, and 441-443).

(Minor comment)

We express our appreciation for the reviewer’s comment on sample size. To estimate sample size in a survival study for time-to-event, we need to input the expected probability of event-free survival at the time point of interest. The main aim of this study was to evaluate lung cancer-related death outcomes in RA patients with or without CPFE. We tried to determine an expected probability of lung cancer-related death or probability of event-free survival based on previous data. As we pointed out in the Introduction, however, there is a lack of studies to examine survival probability in RA patients with lung cancer with or without CPFE. Accordingly, it was not feasible to determine the expected probability in these patient groups. We added an explanation that we could not estimate sample size for this study due to lack of previous data to the Patients and Methods section (lines 195–199). In such a case, the STROBE statement for observational studies in epidemiology gives an example to explain how the sample size was arrived at. We followed this guideline (lines 199–201). We hope our cancer-related mortality data in RA patients with CPFE will be helpful to calculate sample size for future studies.

**Reply to Reviewer 2**

We greatly appreciate the reviewer’s comments about our study overall and the suggestion to compare the duration of RA and CPFE and disease progression and lung cancer-related mortality using the data obtained from this study. To examine the effect of RA duration/progression at baseline on lung-cancer related death outcome, we included these factors into the Fine-Gray competing risks regression analysis. We found that neither RA duration nor Steinbrocker radiological stage (as a measure of RA progression) was associated with lung cancer-related mortality (S3 Table). We also examined the association between RA duration/progression and CPFE complication, and found no significant differences in either RA-related factor among patients with CPFE, those with ILD or emphysema alone, and those without these complications (S2 Table). We included these new data in the Patients and Methods and Results sections of the revised manuscript (lines 226–228, 254–256, and 325–330). New S2 and S3 Tables were also included (lines 493–494 and 496–497). In the present study, we made a definitive diagnosis of CPFE using HRCT scans taken at the diagnosis of lung cancer according to the 2022 official statement of the ATS, ERS, JRS, and ALAT. We were not certain whether the treating physicians had definitively diagnosed these patients as having CPFE before enrollment of our study. Further, disease progression/severity staging of CPFE has not been established at present. Accordingly, we could not determine the duration or progression of CPFE at the time of enrollment of these patients. The definitions and diagnostic criteria of CPFE have recently been proposed by the ATS, ERS, JRS, and ALAT. The statement first proposed to identify CPFE as a syndrome. We hope our study will help physicians increase awareness of the association of CPFE with lung cancer risk and mortality in RA patients. We added these points in the Conclusions section of the revised version (lines 443–448).
