# Supplementary material for: Impact of combined pulmonary fibrosis and emphysema on lung cancer risk and mortality in rheumatoid arthritis: A multicenter retrospective cohort study
Source: PLoS One. 2024 Feb 27;19(2):e0298573. doi: 10.1371/journal.pone.0298573 (PMC10898759; doi:10.1371/journal.pone.0298573)
Supplement: Attachment — Submitted filename: Response to Reviewers.docx [file pone.0298573.s008.docx]

**Response to Reviewers**

We are most grateful to the editor and reviewers for their valuable comments and for the time and energy spent reviewing our manuscript. We have made the requested changes and added new information to the manuscript in response to their insightful comments. All alterations are highlighted in red text in the revised manuscript. We are confident that the manuscript has benefited from the reviewers’ useful comments and suggestions.

Below are point-by-point replies to the reviewers’ comments.

**Reply to Reviewer 1**

We appreciate the reviewer’s understanding that there were several inevitable limitations of our study because this was a retrospective observational study in a real-world clinical setting. Despite these limitations, we obtained clinical evidence for the impact of combined pulmonary fibrosis and emphysema (CPFE) on lung cancer risk and lung cancer-related mortality in patients with rheumatoid arthritis (RA). As this reviewer kindly suggested, we hope that our research will stimulate further investigation of underlying molecular mechanisms in the CPFE-associated pathogenesis of lung cancer in RA.

**Reply to Reviewer 3**

1. We thank the reviewer for the suggestion to compare lung cancer mortality data between RA patients with CPFE and those without this complication because RA *per se* is a well-known risk factor for lung cancer. The subjects of this study were patients with and without RA who had been newly diagnosed with lung cancer in participating institutions. We performed a retrospective follow-up study of these patients until lung cancer-related death, other-cause death, or the end of the study. In the original manuscript, we compared the crude incidence rate of lung cancer-related death as well as the death probability estimated by the cumulative incidence function (CIF) for the three patient groups (CPFE, interstitial lung disease [ILD] or emphysema alone, and the group without these conditions) in all patients as well as separately in RA patients and non-RA patients. The crude incidence rate was highest in the CPFE group in all patients, and the same differences were observed separately in RA and non-RA patients. These data are shown in Table 3 of the manuscript, and we describe these comparison data in the Results section (lines 307–313) of the revised manuscript. Additionally, the CIF mortality estimates over time were significantly worse in the CPFE group. The significant differences among the three patient groups were observed in all patients (*p*<0.001 with Gray’s test), as well as separately in RA and non-RA patients (*p*=0.006 in RA patients and *p*<0.001 in non-RA patients). Please see the Results section (lines 314–322) and Table 3. In the revised manuscript, we added new comparison data of the CIF mortality estimates between RA and non-RA patients grouped according to the HRCT-based CPFE diagnosis. RA patients were estimated to be at higher risk of lung cancer-related death over time compared with non-RA patients in each of the three patient groups (*p*<0.001 with Gray’s test). These comparison data are described in the Results (lines 322–324) and Table 3 footnote.
2. We thank the reviewer for the suggestion to examine the association between RA disease activity and lung cancer-related mortality. In the present study, RA disease activity was well controlled in 68.3% of patients at lung cancer diagnosis (i.e., patients had low disease activity or remission status). There was no significant difference in RA disease activity among the CPFE group, the ILD or emphysema alone group, and the group without these complications. We included this information in the Results section (lines 246 and 261–263) as well as Table 1 and S2 Table of the revised manuscript. To examine the effect of RA disease activity at baseline on lung cancer-related death, we included this variable in the Fine-Gray competing risks regression analysis for RA patients. We found that RA disease activity was not associated with lung cancer-related mortality. We added this information to the Results section (lines 343–348), the Discussion section (lines 380 and 381) as well as in S3 Table.
3. We appreciate the reviewer’s comment regarding the incidence of pulmonary arterial hypertension (PAH). Among patients diagnosed as having CPFE based on HRCT scans taken at the diagnosis of lung cancer, we found 2 patients with PAH. This diagnosis had been made based on data of echocardiography and right heart catheterization (1 in an RA patient and 1 in a non-RA patient). Both patients died during follow-up, and these cases were categorized as lung cancer-related death (oncological emergency events). This information was included in the Results section of the revised version (lines 258–260, 288, and 289).
4. We understand the reviewer’s question regarding whether or not death caused by infectious diseases was included in lung cancer-related death. As mentioned in the “Patients and methods” section of the original manuscript, lung cancer-related death was defined as death caused by cachexia, organ failure, infectious disease (such as pneumonia and sepsis), complications of cancer therapies, or oncological emergencies (line 178–182). In our cohort, 36 patients with lung cancer died from infectious diseases (pneumonia and sepsis; 16 in RA patients and 20 in non-RA patients), and we included these cases in the lung cancer-related death category. We added this information to the Results section of the revised manuscript (lines 287 and 288). As shown in Table 2, there was no other-cause death in RA patients.
5. We are grateful for the reviewer’s suggestion to examine the influence of pulmonary function on mortality in CPFE patients. In the present study, we made a definitive diagnosis of CPFE at study enrollment based on HRCT scans taken at the diagnosis of lung cancer according to the 2022 official guidelines, and we identified 81 CPFE patients (33 in RA patients and 48 in non-RA patients). However, the vast majority of non-RA patients visited the respiratory department of our institution for further examination and treatment of lung cancer. They did not receive pulmonary function tests upon diagnosis of lung cancer. We were not certain whether the general practitioners referring these patients were aware of the presence of CPFE in these non-RA patients or whether they had performed pulmonary function tests for these patients before referral to our respiratory department. Data of percutaneous oxygen saturation [SpO_2_] after a 6-minute walk or equivalent, which is an important parameter for severe pulmonary fibrosis, were available in all RA patients. We found that 47.6% of RA patients showed desaturation with exercise, defined as SpO_2_ <90% during 6-minute walk. The rates of patients with desaturation with exercise were significantly different among the CPFE group, the ILD or emphysema alone group, and the group without these conditions (*p*<0.001). We added this information to the “Patients and methods” section (lines 142 and 143), the Results section (lines 246–248 and 264 –265) as well as in Table 1 and S2 Table.

To examine the effect of desaturation with exercise at baseline on lung-cancer related death outcomes, we included this predictor variable instead of HRCT-based CPFE diagnosis in the multivariable Fine-Gray competing risks regression analysis. HRCT-based CPFE diagnosis was not included in the multivariable analysis with desaturation with exercise because both predictor variables were highly correlated. We found that, like HRCT-based diagnosis, desaturation with exercise was an independent predictive factor for lung cancer-related mortality (adjusted HR 2.58 [95% CI 1.40–4.77]). We included these new data in the Results section (lines 348–354) and the Discussion section (lines 405–410) of the revised manuscript. A new supplementary table was added to the revised version (S4 Table; lines 527 and 528).
